# Ethnic Variation in the Prevalence of Visual Impairment in People Attending Diabetic Retinopathy Screening in the United Kingdom (DRIVE UK)

**DOI:** 10.1371/journal.pone.0039608

**Published:** 2012-06-27

**Authors:** Sobha Sivaprasad, Bhaskar Gupta, Martin C. Gulliford, Hiten Dodhia, Samantha Mann, Dinesh Nagi, Jennifer Evans

**Affiliations:** 1 Laser and Retinal Research Unit, King’s College Hospital NHS Foundation Trust, London, United Kingdom; 2 Division of Health and Social Care Research, King’s College London, London, United Kingdom; 3 Public Health Department, Lambeth NHS Primary Care Trust, London, United Kingdom; 4 Department of Ophthalmology, St. Thomas Hospital NHS Foundation Trust, London, United Kingdom; 5 Endocrinology and Diabetes, Mid Yorkshire NHS Trust, Wakefield, Yorkshire, United Kingdom; 6 Department of Epidemiology, London School of Hygiene and Tropical Medicine, London, United Kingdom; University of Swansea, United Kingdom

## Abstract

**Purpose:**

To provide estimates of visual impairment in people with diabetes attending screening in a multi-ethnic population in England (United Kingdom).

**Methods:**

The Diabetic Retinopathy In Various Ethnic groups in UK (DRIVE UK) Study is a cross-sectional study on the ethnic variations of the prevalence of DR and visual impairment in two multi-racial cohorts in the UK. People on the diabetes register in West Yorkshire and South East London who were screened, treated or monitored between April 2008 to July 2009 (London) or August 2009 (West Yorkshire) were included in the study. Data on age, gender, ethnic group, visual acuity and diabetic retinopathy were collected. Ethnic group was defined according to the 2011 census classification. The two main ethnic minority groups represented here are Blacks (“Black/African/Caribbean/Black British”) and South Asians (“Asians originating from the Indian subcontinent”). We examined the prevalence of visual impairment in the better eye using three cut-off points (a) loss of vision sufficient for driving (approximately <6/9) (b) visual impairment (<6/12) and (c) severe visual impairment (<6/60), standardising the prevalence of visual impairment in the minority ethnic groups to the age-structure of the white population.

**Results:**

Data on visual acuity and were available on 50,331individuals 3.4% of people diagnosed with diabetes and attending screening were visually impaired (95% confidence intervals (CI) 3.2% to 3.5%) and 0.39% severely visually impaired (0.33% to 0.44%). Blacks and South Asians had a higher prevalence of visual impairment (directly age standardised prevalence 4.6%, 95% CI 4.0% to 5.1% and 6.9%, 95% CI 5.8% to 8.0% respectively) compared to white people (3.3%, 95% CI 3.1% to 3.5%). Visual loss was also more prevalent with increasing age, type 1 diabetes and in people living in Yorkshire.

**Conclusions:**

Visual impairment remains an important public health problem in people with diabetes, and is more prevalent in the minority ethnic groups in the UK.

## Introduction

Visual impairment adds to the burden of several other microvascular and macrovascular complications in people with diabetes, threatens independence and compromises quality of life [1]. Estimates of blindness in diabetes from the United Kingdom (UK) are mainly based on audits of certifications of visual impairments which are not population-based estimates [2]–[9]. Other sources of data on diabetes-related visual impairment in the UK are derived from multipurpose health surveys aimed at a specific population [10]–[12] or limited to the Caucasian population [13]–[17].

The demographic composition of the UK is changing with a rise in the ageing population and most of its metropolitan cities now have an ethnically diverse composition. The prevalence of diabetes is disproportionately higher in the non-Caucasian ethnic groups [18]. It is clear that better glycaemic and blood pressure control among people with diabetes has resulted in a reduction of adverse outcomes of diabetes as observed in previous studies conducted in the United States and the UK [19], [20]. A recent report from the UK also suggested that diabetic retinopathy (DR) is no longer the most common cause of visual impairment in the working age-group [2]. It is important to obtain current population-based data on low vision and severe visual impairment to understand the impact of these changes on the healthcare burden in the UK.

These contemporary data will also provide baseline data to assess the impact of diabetic retinopathy screening and forth-coming new treatment options for diabetic macular oedema.

In 2010, the Association of Public Health Observatories (AHPO) prevalence model estimated that there were approximately 3 million people aged 16 years or above with undiagnosed and diagnosed diabetes in England [21]. The Quality and Outcomes Framework (QOF) requires each general practice to maintain a register for all people aged 17 years and over with diabetes mellitus, which specifies whether the person has Type 1 or Type 2 diabetes. These data are uploaded to the diabetic register maintained by the DR screening programme. The UK has one of the most developed and quality assured DR screening programmes in the world with a population coverage ranging from 80–95% [22]. People with sight threatening disease are referred to hospital retinal services for timely management. It is therefore feasible to obtain current epidemiological data from a large nationally representative cohort on visual impairment in people with diagnosed diabetes.

The aim of the Diabetic Retinopathy in Various Ethnic groups in UK (DRIVE UK) is to provide a cross-sectional analysis of visual acuity and diabetic retinopathy in people with diabetes attending for screening in two ethnically diverse regions in the UK.

## Methods

### Ethics Statement

The study adhered to the Declaration of Helsinki and was approved by the Chair of the Research Ethics Committee of King’s College Hospital NHS foundation trust and the London School of Hygiene and Tropical Medicine. Written consent from patients was not required for this project as only anonymized data from the regional diabetes registers were analysed.

### Study Methodology

The Diabetic Retinopathy in Various Ethnic groups in the UK (DRIVE-UK) study is a cross-sectional analysis of two databases containing data on a total of 57,144 people with diagnosed diabetes in West Yorkshire (registered subjects with family practices in Wakefield and North Kirklees) and South East London (registered subjects with family practices in Lambeth, Southwark and Lewisham). These regions provide populations representative of the multiethnic inhabitants in the UK. The minority ethnic groups constitute 7.6% of the population of the UK with most settled in metropolitan cities [23]. The largest minority groups can be categorised into Blacks (Black African, Black Caribbean, any other Blacks) and South Asians (descent from India, Pakistan and Bangladesh and SriLanka). South East London has an ethnic mix of Blacks, South Asians, mixed and other groups while South Asians are the predominant minority ethnic group in West Yorkshire.

### Data Collection

The majority (>95%) of the population in the UK are registered with a family practice. Since the introduction of the Quality and Outcomes Framework (QoF) all people diagnosed with diabetes are placed on a register with their local family practices so that systematic care can be provided. Data on age, gender and type of diabetes and ethnicity are uploaded from practice diabetes registers into a single collated list at the local DR screening programmes. The digital photographic diabetic retinopathy screening programmes in the UK are well-established and 100% of people with diabetes are offered screening and the uptake rates are at least 70%, with most screening programmes achieving over 85% annually. It is therefore possible to analyse population-based data on visual acuity on all people diagnosed with diabetes who take up these services. Both programmes included in this study provide a reasonably comprehensive coverage of diabetics in the respective regions -95% in West Yorkshire and 81% in South East London.

Individuals that require specialist input or treatment are referred to specified hospital eye services for further management. Those who are exempt from screening include those who are excluded because they have no perception of light or suspended as they are under the care of an ophthalmologist in secondary care either due to sight threatening disease or because the fundus cannot be assessed by digital photography. From the screening databases in the two regions, data were collected on age, gender, ethnicity, physician-reported type of diabetes, visual acuity and grade of retinopathy. Similar data on people exempted from screening were obtained from the certificates of visual impairment and clinical records of hospital eye services. The cross-sectional data were collected in August 2009 from West Yorkshire and September 2009 in South East London and the data from the latest episode of screening or eye clinic appointment within the last 15 months of the data collection date was used.

### Ethnicity Data

Data on ethnicity were self ascertained using the 16 categories recorded in the 2001 census (table 1) [23].

**Table 1:** Ethnic group classification: census classification.

White	English/Welsh/Scottish/Northern Irish/Irish; Gypsy or Irish Traveller; Any other White background.
Mixed/multiple ethnic groups	White and Black Caribbean; White and Black African; White and Asian; Any other Mixed/multiple ethnic background.
Asian/Asian British	Indian; Pakistani; Bangladeshi; Chinese; Any other Asian background.
Black/African/Caribbean/Black British	African; Caribbean; Any other Black/African/Caribbean background.
Other ethnic group	Arab; Any other ethnic group

South Asian in this study is defined as Indians, Pakistanis, Bangladeshi and Srilankan.

### Visual Acuity Testing

The protocols for recording visual acuity in both screening programmes were as recommended by the National Screening Committee [24]. Presenting visual acuity for distance was recorded for each eye before dilating the pupils for fundus photography. This was measured with the participant wearing their “walk-in” optical correction (i.e. spectacles or contact lenses) using Early Treatment of Diabetic Retinopathy Study (ETDRS) charts.^24^ If no letters were read at 2 metres, visual acuity was assessed as counting fingers, hand movements, perception of light, or no perception of light. The visual acuity of the better eye was used for all analyses. Age-standardisation of the prevalence of visual impairment in the minority ethnic groups was done based on the age-structure of the white population.

### Definition of Visual Impairment

We examined the prevalence of visual impairment in the better eye using three cut-off points: (a) loss of vision sufficient for driving (approximately <6/9) (b) visual impairment (<6/12) and (c) severe visual impairment (<6/60).

### Screening and Grading of Diabetic Retinopathy

A mydriatic 2-field digital photography, one centred on the optic disc and the other on the macula was carried out on all diabetic people attending for screening. Trained graders carried out a full disease grade on all image sets; a different grader then independently assessed 10% of the no-disease sets and all-disease image sets. If there was a difference of opinion about referral, the images were arbitrated by an ophthalmologist. The grading of DR was done according to the English Retinopathy Minimum grading classification [25]. As some people with diabetes were followed up in secondary care, the data on the grades of DR for these people were obtained from hospital records.

### Statistical Analyses

Data were analysed using STATA version 12 (StataCorp, Texas, USA). Analyses were largely descriptive and included calculating the percentage of people visually impaired according to different definitions in different population subgroups with associated 95% confidence intervals. We also did three logistic regression models examining risk factors for visual impairment (<6/9, <6/12 and <6/60) compared to those who are not in that case-group. We included terms for age in 10-year age groups, gender (male/female), location (London/Yorkshire), type of diabetes (I/II) and ethnic group (Caucasian, African/Afro-Caribbean and South Asian) and retinopathy grades (any DR, sight threatening DR and maculopathy) in these models.

## Results

The QOF data from general practitioners indicated that the numbers of people with diagnosed diabetes within the specified areas in West Yorkshire (North Kirklees and Wakefield) from June 2008-August 2009 and South East London (Lambeth, Lewisham and Southwark) from July 2008-September 2009 were 20,878 and 36,266 respectively. Of these 18,558 (88.9%) and 31,773 (87.6%) respectively had data on visual acuity and diabetic retinopathy status during the data collection period in West Yorkshire and in South East London and the data from the latest episode of screening or eye clinic appointment within this time period was used. In West Yorkshire, the numbers of people with no data included those that were medically unfit for screening (268), moved out of area (41), denied having diabetes (5) and had opted out of the screening programme or screened elsewhere (82). A further 1001 people had no ethnicity data, 11 were other types of diabetes and no data was available from recent hospital records on 912 during this period. In South East London, the numbers of people with no data included those that were medically unfit for screening or institutionalised (235), moved out of area (142), denied having diabetes (5), were under 12 years of age (10) and had opted out of the screening programme or screened elsewhere (244). A further 2710 had no ethnicity data and there were 1147 missing records from hospital eye services or general practices that did not participate in uploading data to the screening programmes.

Table 2 shows the characteristics of the study population. The mean age of the study population (n = 50,331) was 62.0 years with 59.7% being aged 60 years or older. There were similar numbers of men and women. Overall, 34% of this study population were composed of minority ethnic groups. The minority ethnic groups in South East London and West Yorkshire comprised 47.1% and 12.6% of the total people with diabetes on the registers respectively with South Asians predominating in West Yorkshire and Blacks in South East London: Most people (93%) were diagnosed with Type 2 diabetes. The prevalence of diagnosed diabetes in this study is 4.1% with similar rates of diagnosed diabetes between the three ethnic groups, suggesting that undiagnosed diabetes and/or uptake of retinal screening remain an issue especially in the minor ethnic groups in the UK.

**Table 2:** Characteristics of the study population.

	South East LondonN = 31,773	West YorkshireN = 18,558	TotalN = 50,331
Mean age (SD)	61.1 (14.7)	63.6 (14.7)	62.0 (14.8)
	*N(%)*	*N(%)*	*N(%)*
Age <30	757 (2.4)	475 (2.6)	1232 (2.5)
Age 30–39	1674 (5.3)	716 (3.9)	2390 (4.8)
Age 40–49	4666 (14.7)	1801 (9.7)	6467 (12.9)
Age 50–59	6780 (21.3)	3419 (18.5)	10199 (20.3)
Age 60–69	7573 (23.8)	5024 (27.1)	12597 (25.0)
Age 70–79	7234 (22.8)	4771 (25.8)	12005 (23.9)
Age 80+	3087 (9.7)	2324 (12.5)	5411 (10.8)
Male	16307 (51.3)	10318 (55.6)	26625 (52.9)
Diabetes Type I	2112 (6.7)	1211 (6.5)	3323 (6.6)
Diabetes Type II	29630 (93.4)	17332 (93.5)	46962 (93.4)
White	16815 (52.9)	16194 (87.4)	33009 (65.6)
Black	8227 (25.9)	149 (0.8)	8376 (16.7)
South Asian	1478 (4.7)	2040 (11.0)	3518 (7.0)
Mixed	2631 (8.3)	55 (0.3)	2686 (5.3)
Other	2622 (8.3)	91 (0.5)	2713 (5.4)

Table 3 shows the distribution of vision in the study cohort by various demographic factors.

**Table 3:** Distribution of visual acuity by various demographic factors.

Vision in thebetter eyeN(%)	Total	South EastLondon	WestYorkshire	Type Idiabetes	Type IIdiabetes	Men	Women	WhiteEuropean	African/Afro-Caribbean	SouthAsian
Total	50,330(100)	31,773(100)	18,557(100)	3,323(100)	46,961(100)	26,624(100)	23,691(100)	33,009(100)	8,376(100)	3,518(100)
> = 6/9	46,543(92.5)	29,860(94.0)	16,683(89.9)	3,183(95.8)	43,317(92.2)	24,902 (93.5)	21,629(91.3)	30,575(92.6)	7,702(92.0)	3,153(89.6)
<6/9–6/12	2,088(4.2)	1,036(3.3)	1,052(5.7)	75(2.3)	2,012(4.3)	957(3.6)	1,129(4.8)	1,345(4.1)	364(4.4)	196(5.6)
<6/12–6/18	873(1.7)	412(1.3)	461(2.5)	34(1.0)	838(1.8)	379(1.4)	494(2.1)	568(1.7)	151(1.8)	88(2.5)
<6/18–6/60	631(1.3)	342(1.1)	289(1.6)	26(0.8)	604(1.3)	285(1.1)	345(1.5)	401(1.2)	114(1.4)	65(1.9)
<6/60	195(0.4)	123(0.4)	72(0.4)	5(0.2)	190(0.4)	101(0.4)	94(0.4)	120(0.4)	45(0.5)	16(0.5)

Table 4 combines the data in the entire study population according to various cut-points of visual acuity. Overall, 3787 (7.5%, 95% confidence intervals (CI) 7.3% to 7.8%) of the people with diabetes attending screening were not eligible for driving based on their presenting visual acuity and 1699 (3.4%, 3.2% to 3.5%) were visually impaired (<6/12) and 195 (0.4%, 0.33% to 0.44%) severely visually impaired (<6/60). The other cut-points are provided for comparison purposes, for example, cut-points (< = 6/18 and < = 6/60) are the cut-points used by the National Screening Committee. The table also shows the prevalence of visual impairment by ethnic group, with the prevalence estimates for the minority ethnic groups directly age-standardised to the age-structure of the white population. There was a trend such that people of black African/African-Caribbean origin, diagnosed with diabetes and attending for screening, had a higher risk of visual loss compared to their white European counterparts; people of South Asian origin had a higher risk of visual impairment compared to black African/Afro-Caribbean people on the register. This trend was observed for each visual acuity cut-point, but since there were relatively few cases of severe visual impairments, ethnic differences in severity of visual impairment were not statistically significant.

**Table 4:** Prevalence of visual impairment in people with diabetes.

Visual impairment*	Ethnic group	Prevalence		Age-standardised prevalence**	
		N	%	%	95 % CI
<6/9 (approximate cut-point for driving vision)	All ethnic groups combined	3787	7.5	–	–
	White European	2434	7.4	7.4	7.1,7.6
	African/Afro-Caribbean	674	8.0	9.7	9.0,10.4
	South Asian	365	10.3	14.7	13.3,16.2
<6/12 (< = 6/18) (visual impairment)	All ethnic groups combined	1699	3.4	–	–
	White European	1089	3.3	3.3	3.1,3.5
	African/Afro-Caribbean	310	3.7	4.6	4.0,5.1
	South Asian	169	4.7	6.9	5.8,8.0
<6/18	All ethnic groups combined	826	1.6	–	–
	White European	521	1.6	1.6	1.4,1.7
	African/Afro-Caribbean	159	1.9	2.3	1.9,2.7
	South Asian	81	2.3	3.1	2.4,3.9
< = 6/60	All ethnic groups combined	313	0.62	–	–
	White European	194	0.59	0.59	0.51,0.67
	African/Afro-Caribbean	67	0.80	0.98	0.73,1.22
	South Asian	27	0.77	1.22	0.71,1.74
<6/60 (severe visual impairment)	All ethnic groups combined	195	0.39	–	–
	White European	120	0.36	0.36	0.30,0.43
	African/Afro-Caribbean	45	0.54	0.64	0.44,0.83
	South Asian	16	0.46	0.77	0.34,1.20

* Vision in the better eye. Study cut-points (<6/9, <6/12 and <6/60) given as well as cut-points for comparison purposes (<6/18 WHO visual impairment and < = 6/60 NSC criteria).

** Standardised to the age-structure of the Caucasian population.

*** In this dataset: <6/12 was the same numerically as < = 6/18 (NSC criteria).

White European n = 32,989 African/Afro-Caribbean n = 8375, South Asian n = 3510.

**Table 5:** Logistic regression analysis for each of the visual impairment categories.

Odds ratio (95% CI)	Less than 6/9(n = 3787)	Less 6/12(n = 1699)	Less than 6/60(n = 195)
Age (per year age)	1.065 (1.062, 1.068)	1.073 (1.068,1.078)	1.082 (1.068,1.096)
Men	1	1	1
Women	1.27 (1.19,1.36)	1.25 (1.13,1.38)	0.90 (0.67,1.19)
South East London (DECS)	1	1	1
West Yorkshire (DRSS)	1.94 (1.79, 2.11)	1.74 (1.55,1.96)	1.02 (0.74, 1.43)
Type I diabetes	1	1	1
Type II diabetes	0.50 (0.41, 0.60)	0.44 (0.33, 0.57)	0.63 (0.26, 1.56)
White	1	1	1
Black	2.00 (1.80, 2.22)	1.98 (1.70, 2.29)	2.02 (1.38, 2.97)
Asians	2.21 (1.96, 2.50)	2.33 (1.96, 2.78)	2.00 (1.18, 3.41)
Other	1.39 (1.21, 1.59)	1.27 (1.04, 1.55)	0.98 (0.55, 1.75)

Vision in better eye; Adjusted for all other factors in the table.

Logistic regression analyses (table 5) showed that the risk of visual impairment in all 3 categories (visual impairment for driving <6/9, visual impairment <6/12 and severe visual impairment <6/60) increased with increasing age but was not consistently associated with gender. The risk of visual impairment in type 1 diabetes was twice that in type 2 diabetes. Minority ethnic groups (both South Asians and Blacks) were twice as likely to be visually impaired in all 3 categories of definitions compared to their white counterparts. A regional variation in visual impairment was also observed with the West Yorkshire cohort having more visual impairment than South East London people.

Table 6 shows the association between retinopathy and visual impairment in the right eye (similar results for left eye, data not shown). Visual impairment increased with increasing signs of retinopathy and maculopathy in the eye. Logistic regression analyses, adjusting for age, sex, location and ethnic group, showed that people with proliferative diabetic retinopathy in their right eye had 13.8 times increased odds of having vision less than 6/9, 13.2 increased odds of having vision less than 6/12 and 11.4 increased odds of having vision less than 6/60, in their right eye, compared to people with no retinopathy in their right eye.

**Table 6:** Vision and diabetic retinopathy.

Retinopathyin right eye	No Retinopathy	Mild and moderatenon-proliferativeRetinopathy	Severe non-proliferativeRetinopathy	Proliferativediabeticretinopathy	Diabeticmaculopathy
Vision in right eye	N (%)	N (%)	N (%)	N (%)	N (%)
6/9 or better	30,755 (86.4)	10,129 (79.6)	459 (70.0)	431 (34.6)	1,708 (54.6)
<6/9–6/12	2,001 (5.6)	1181 (9.3)	87 (13.2)	182 (14.6)	480 (15.4)
<6/12–6/18	1,259 (3.5)	690 (5.4)	42 (6.4)	235 (18.9)	355 (11.4)
<6/18–6/60	1,080 (3.0)	554 (4.4)	59 (9.0)	233 (18.7)	402 (12.9)
<6/60	494 (1.4)	179 (1.4)	13 (2.0)	166 (13.3)	179 (5.7)
	35,589 (100)	12,733 (100)	660 (100)	1,247 (100)	3,124 (100)

## Discussion

Approximately 3.4% of people diagnosed with diabetes and attending for screening were visually impaired (vision in the better eye of <6/12) and 0.39% were severely visually impaired. People of Asian and African descent were twice at risk of visual impairment in categories of driving vision, low vision and severe visual impairment compared to white people.

This study highlights that even though people with diabetes participate in screening of DR using digital fundus photography, visual impairment remain a significant public health problem in the UK. Current digital photographic retinal screening for DR may not be sufficient to reduce the overall prevalence of visual impairment in diabetes due to the low contribution of DR to visual impairment. Uncorrected refractive error, cataract and glaucoma are more common in people with diabetes than the non-diabetic population and contribute more to visual impairment than diabetic retinopathy [15], [26], [27]. Although people with diabetes are offered free eye-sight test in UK, the spectacles are often unaffordable. Studies from around the world also indicate that the threshold to correct one’s refraction varies considerably between ethnic groups. Furthermore, some of these ocular co-morbidities such as cataract in South Asians and glaucoma in the Afro-Caribbean population are more prevalent in certain ethnic groups [27]–[29]. These factors may explain the ethnic differences in visual impairment observed in this study.

Loss of vision is uncommon in studies on DR from Iceland. This comparison with the Icelandic sample may not be ideal as the population is relatively stable, exclusively Caucasian population with few migrants and the provision for eyeglasses is covered. The Icelandic population is also carefully screened for diabetes mellitus and has been provided with regular screening for DR since 1983 [30], [31]. It is still too early for the national systematic screening programme for DR in UK to produce a positive impact on the prevalence of visual impairment. Only time will tell if such findings could be translated to a multiracial and mobile population.

The analyses of severity of DR between ethnic groups in this study showed that the minority groups are also twice as likely to have sight threatening DR is also twice in compared to the Caucasian counterpart [32]. People with proliferative DR and persistent maculopathy despite laser treatment are especially vulnerable to visual impairment. These observations are consistent with the findings of the UKADS study on South Asians that showed that the risk of sight threatening retinopathy is significantly higher than Caucasians and that this disparity could partly be explained by differential susceptibility to systemic risk factors [33]. Previous studies evaluating ethnic differences in certifications of visual impairment also showed similar results with the proportion of South Asians who are registered blind due to DR being three times that of the Caucasians in the UK [6], [8]. So, it is likely that DR may also contribute to the higher prevalence of visual impairment in this minority population. There is very limited data on visual impairment in the Blacks with diabetes in the UK.

The other risk factors in minor ethnic groups include an earlier age of onset of diabetes and poorer health care utilization rates [34]. These findings are of concern, as subjects who are at highest risk seem to have poorer outcomes. Previous studies on education levels and socio-economic status have shown that people with low income and those with lower levels of education are at higher risk of visual impairment, cataract and PDR [35]. The effect of race on ocular diseases was highlighted as early as 1990 in the United States in the Baltimore Eye Survey that showed that people of African descent had, on average, a twofold greater prevalence of blindness and visual impairment compared to Caucasians [36]. This effect of race was reduced after adjustment of the socio-economic factors. Socio-economic deprivation is likely to play a role in outcome and may be a limiting factor in this study. The rates of visual impairment in high income countries are going down and this is not mirrored in the UK as yet. Perhaps the stable rates in the UK may be in part attributable to the continued influx of immigrants with diabetes which, in turn negate the benefits made to reduce the risk of DR among long standing residents whose diabetes has been carefully managed for some time. So, further research into individual level data focussing on this aspect in the UK is warranted.

This study also confirmed that increasing age is a risk factor for visual impairment in diabetes. Although DR is the commonest cause of visual impairment in the working age-group, relative to other causes, people aged 65 years and older with diabetes are three times more likely to be visually impaired (in all 3 categories- driving vision, low vision and severely visually impaired) compared to those between 16–64 years. It may be postulated that these figures may only reflect the increasing prevalence of diabetes in the older people who mainly suffer from other causes of visual impairment especially cataract and age related macular degeneration. However, Bunce et al observed that the rates of registration (both low vision and blindness) due to DR in the elderly have increased significantly in the last two decades [3]. Although this rise is often attributed to increased public, professional and political awareness of certifications and support provided as part of the VISION 2020 strategy, this study highlights the fact that visual impairment is definitely a significant public health issue in the older population with diabetes.

Visual impairment also occurs more frequently in people with type 1 diabetes compared to type 2 diabetes. Screening and timely management of DR has been shown to reduce the risk of visual impairment in people with type 1 diabetes [37]. A similar reduction in type 2 diabetes is more difficult to achieve unless diabetes is diagnosed early and appropriate interventions are given promptly [38].

We have focused on presenting vision–that is, visual acuity as used in everyday life by the people taking part in DR screening. This measure of visual impairment is the most relevant for public health purposes [11]. Despite that our prevalence may be an underestimate of visual impairment in people with diabetes because the study did not include non-attenders to the screening programmes and associated eye clinics. Our previous study on the South East London cohort indicated that screening uptake rates were particularly poor among the young adults aged 18–34 years and those aged 85 years or greater [39].

Another limitation of our study is that approximately 30% of the non-participants were those referred to hospital eye services for referable DR or unclassifiable retinopathy using digital photography due to ungradable images. So it is likely that the actual prevalence of visual impairment may again be underestimated as there is variability in assessing DR severity grade between screening programmes and secondary care. However, the results of our study compare well with other studies that examined subjects from the local diabetic retinopathy screening programmes in predominantly Caucasian-inhabited regions in the UK. The Liverpool Eye study in 1999 observed that 3.4% had visual acuity of ≤6/24 and 0.8% had visual acuity of ≤6/60 [40]. Prasad et al noted that the prevalence of low vision and blindness as per WHO classification in Wirral were 2% and 0.75% in 2000 [14] and in Gloucestershire, Scanlon et al reported these to be 2.9% and 0.45% respectively in 2008 [15]. It is useful to note that whatever be the source of data collection (survey of DR screening database or register of certifications of visual impairment), the prevalence of visual impairment in people with diabetes has been stable in the last decade [3], [5]–[8], [14], [15].

In summary, this study highlights the ethnic –specific prevalence of visual impairment in the UK. The ethnic differences may be due to patient level characteristics such as genetic differences; differences in control of risk factors of diabetes; differences in knowledge of complications of diabetes; or service level characteristics including access to care and treatment outcomes or probably a combination of these factors.

With the increasing population, the demographic right shift of the population and the emerging racial-mix in most cities in the UK, [18], [23] it is important to identify the causes of visual impairment in people with diabetes before any strategic recommendations can be made in relation to resource allocation.
